# Preparation of geometrically highly controlled Ga particle arrays on quasi-planar nanostructured surfaces as a SCALMS model system†

**DOI:** 10.1039/d2ra07585g

**Published:** 2023-01-27

**Authors:** André Hofer, Nicola Taccardi, Michael Moritz, Christoph Wichmann, Sabine Hübner, Dominik Drobek, Matthias Engelhardt, Georg Papastavrou, Erdmann Spiecker, Christian Papp, Peter Wasserscheid, Julien Bachmann

**Affiliations:** a Friedrich-Alexander-Universität Erlangen-Nürnberg (FAU), Department of Chemistry and Pharmacy, Chemistry of Thin Film Materials, IZNF Cauerstr. 3 91058 Erlangen Germany julien.bachmann@fau.de; b Friedrich-Alexander-Universität Erlangen-Nürnberg (FAU), Department Chemical and Biological Engineering, Institute of Chemical Reaction Engineering (CRT) Egerlandstr. 3 91058 Erlangen Germany; c Friedrich-Alexander-Universität Erlangen-Nürnberg (FAU), Department of Chemistry and Pharmacy, Chair of Physical Chemistry II Egerlandstr. 3 91058 Erlangen Germany; d Friedrich-Alexander-Universität Erlangen-Nürnberg (FAU), Institute of Micro- and Nanostructure Research (IMN) & Center for Nanoanalysis and Electron Microscopy (CENEM), IZNF Cauerstr. 3 91058 Erlangen Germany; e Universität Bayreuth, Chair of Physical Chemistry II Universitätsstr. 30 95447 Bayreuth Germany; f Angewandte Physikalische Chemie, FU Berlin Arnimalle 22 14195 Berlin Germany; g Forschungszentrum Jülich GmbH, Helmholtz Institute Erlangen-Nürnberg for Renewable Energy (IEK-11) Cauerstr. 1 91058 Erlangen Germany

## Abstract

This study establishes a preparative route towards a model system for supported catalytically active liquid metal solutions (SCALMS) on nanostructured substrates. This model is characterized by a uniquely precise geometrical control of the gallium particle size distribution. In a SCALMS system, the Ga serves as a matrix material which can be decorated with a catalytically active material subsequently. The corresponding Ga containing precursor is spin-coated on aluminum based substrates, previously nanostructured by electrochemical anodization. The highly ordered substrates are functionalized with distinct oxide coatings by atomic layer deposition (ALD) independently from the morphology. After preparation of the metal particles on the oxide interface, the characterization of our model system in terms of its geometry parameters (droplet diameter, size distribution and population density) points to SiO_2_ as the best suited surface for a highly controlled geometry. This flexible model system can be functionalized with a dissolved noble metal catalyst for the application chosen.

## Abstract 2

2D SCALMS model system preparation offering a high degree of geometric control of the Ga matrix particles by combination of nanostructured surfaces with independent adjustment of the substrates' surface chemistry in a thermal decomposition approach.

## Graphical abstract

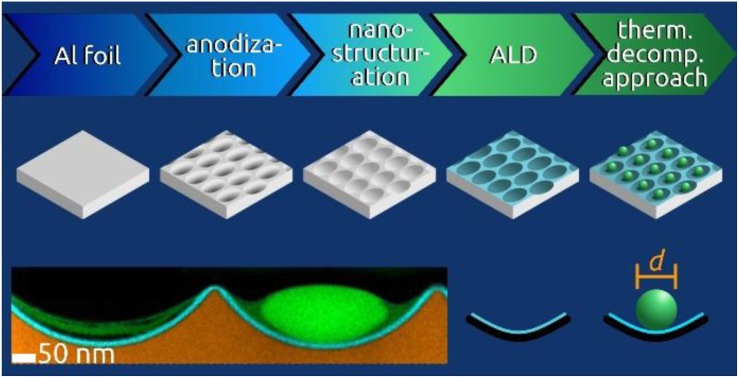

## Introduction

Liquid metals (LM) have recently emerged in catalysis due to their unique properties: existing in the liquid phase at a wide range of process temperature, low toxicity, negligible vapor pressure, high electric and thermal conductivity (improving heat transfer), low viscosity compared to heavy oils, and, perhaps most importantly, elimination of deactivation by coking and high ability of alloying.^1^ Here, the liquid material itself acts as the catalyst for the chemical transformation. This principle can be extended to systems that increase the practical applicability in two ways. If firstly, the catalytically active element is dissolved in the liquid metal to reduce its total amount and secondly the liquid alloy is placed on a porous solid substrate for ease of handling, one obtains a so-called SCALMS, a supported catalytically active liquid metal solution. In such systems, the dynamics of the catalyst element in a dynamic liquid matrix protects it from undesired deactivation and opens the possibility of simple catalyst modifications (dissolution of distinct catalysts or mixtures). However, the experimental determination of fundamental kinetic parameters such as turnover frequencies (per noble metal center) has not been possible so far. This limitation is due to the physical state of dispersion, in which an extremely broad distribution of metal particle sizes exists with a porous glass support of complex geometry. In this work, we present a preparative approach to resolve this issue experimentally. We establish a system of SCALMS particles with a narrow size distribution placed on a quasi-planar structure. We also pay attention to preventing the massive oxidation of the air-sensitive metallic phase. Furthermore, we prove the generality of the method by applying it to four different types of metal oxide substrates, and demonstrate a marked influence of the support chemistry on the particles.

## Experimental

### Materials

Standard chemicals (phosphoric acid, hydrochloric acid, perchloric acid, chromium trioxide, ethanol, toluene) were purchased from Carl Roth and used without further purification. Ethanol was distilled in a rotary evaporator and aluminum plates (99.99%) were purchased from SmartMembranes.

### Substrate preparation

The nanostructured substrates with a defined indentations diameter were prepared by a one-step anodization procedure.^2–4^ For this process, ultrapure aluminum plates (area approx. 5 cm^2^) were placed in purpose-built two-electrode cells based on a PVC beaker serving as container of the electrolyte with four circular openings, closed with a lid equipped with a silver wire mesh acting as counter-electrode and a mechanical stirrer. The Al sheets were held between an O-ring and a thick copper plate serving as the second electrical contact. Prior to anodization, the Al plates were electropolished in an ice-cooled solution of perchloric acid and ethanol (HClO_4_ : EtOH, 1 : 3 v/v) under stirring under a potential of +20 V applied for 7 min (DC Power Supply, PS-1302 D by Voltcraft). The beakers were subsequently rinsed, cooled, filled with 100 mL of electrolyte (aqueous 0.5 wt% H_3_PO_4_), thermally isolated and anodized under stirring at an applied potential of +195 V for 1 h at 0 °C (power supply, EA-PS-8360-10 DT or EA-PS-8360-10 T by EA-Elektro Automatik). Adjacent, the H_3_PO_4_ concentration was increased to 1.0 wt% and the anodization continued for another 23 h. The sacrificial disordered oxide layer obtained due to anodization was removed by a bath in chromic acid (0.18 M CrO_3_ in 6 wt% H_3_PO_4_) at 45 °C for 24 h (VENTI-Line oven by VWR) yielding well-ordered indentations in the Al plates.

### Atomic layer deposition (ALD)

In order to adjust the surface chemistry independently from the geometry of the substrate, various metal oxide thin films were deposited on the nanostructured substrate using ALD in a commercial Gemstar-XT-6 ALD reactor from Arradiance. In all cases, the nominal thickness of the metal oxide film was determined on native SiO_2_ wafers by spectroscopic ellipsometry with a SENPro from SENTECH. SiO_2_: 3-aminopropyltriethoxysilane (APTES) heated to 70 °C, water and ozone, generated by an ozone generator model BMT 803N, were used as precursors, whereas the reaction chamber was maintained at 170 °C.^5^ MgO: di(cyclopentadienyl)magnesium heated to 57 °C and water were used as precursors, whereas the reaction chamber was maintained at 150 °C.^6^ TiO_2_: titanium(iv) isopropoxide heated to 70 °C and water were used as precursors, whereas the reaction chamber was maintained at 150 °C.^7^ Al_2_O_3_: trimethylaluminium and water (both unheated) were used as precursors, whereas the reaction chamber was maintained at 120 °C.^8^

### Gallium particle generation

H_3_Ga(quinuclidine) was synthesized as described by Atwood *et al.*^9^ All following steps were performed under inert atmosphere in order to prevent contact with moisture or oxygen and undesired oxidation of the highly sensitive Ga particles. A solution of trihydridogallium(quinuclidine) (1.0 M, 50 μL) in dry toluene was dropped on the nanostructured substrates (coated with metal oxide) and spin-coated for 30 s at 3000 rpm. The remaining solvent was evaporated and the Ga complex thermally decomposed on a hot plate at 300 °C within 60 s forming metallic Ga particles and volatile by-products.

### Catalyst deposition by galvanic replacement

The Ga particles were decorated with noble metals, which are the catalytically active material in this SCALMS model system, by galvanic replacement reactions in an aqueous/IPA solution in air. Aqueous solutions of ammonium tetrachloropalladate(ii) (NH_4_)_2_[PdCl_4_] with a Pd content of 1 mg mL^−1^ and chloroplatinic acid H_2_PtCl_6_ with a Pt content of 4.4 mg mL^−1^ were used as precursor and diluted with isopropanol to the noble metal to gallium ratio stated in the text. After the exchange was completed (pale yellow Pt solution turned colorless), the sample was rinsed with solvent and dried in a N_2_ stream.

### Analyses

The geometry, morphology and EDX mapping of the SCALMS model systems were investigated using a JSM-F100 field-emission scanning electron microscope by JEOL, the composition by a scanning electron microscope JEOL JSM 6400 equipped with a LaB_6_ cathode combined with an energy-dispersive X-ray (EDX) detector from SAMx. The crystal structure was analyzed by X-ray diffraction (GI-XRD) in grazing-incidence geometry using a Bruker D8 Advance equipped with a Cu Kα source and LynxEye XE detector.

STEM imaging, more specifically with high-angle annular dark-field (HAADF), and STEM EDX analysis were performed in a double-aberration corrected FEI Titan Themis^3^ 300 transmission electron microscope with an accelerating voltage of 300 kV. For this purpose, a focused ion beam was used in an FEI Helios NanoLab 660 SEM/FIB dual beam system to prepare a thin cross-sectional lamella through a selected region containing several Ga particles. To this end, the area was covered with a protective carbon layer using the gas injection system (GIS), cut free from bulk material using the FIB, and transferred to a lift-out TEM grid (PELCO Indicator) using the so-called lift-out technique. Subsequently, the lamella was thinned down by FIB milling from both sides to a thickness of less than 100 nm to allow local and precise STEM-EDX analyses. The EDX measurements were performed at a convergence angle of 15.7°, a dwell time of 50 μs and a current of about 400 pA.

XPS measurements were carried out with a custom-built NAP-XPS instrument from the Institute of Physical Chemistry II in Erlangen. The measurement chamber is equipped with a Specs NAP XR50 X-ray tube with a dual Al/Mg anode. The hemispherical photoelectron analyzer is a modified Omicron EA 125 U7, which is separated from the measurement chamber by multiple pumping stages. More information about the XPS instrument can be found in Pantförder *et al.*^10^ Sample transfer was performed using an airtight and mobile stainless steel UHV system equipped with a transfer arm, a Pirani gauge for pressure measurement, and a fine dosing valve for vacuum pumping. The compact system is closed with a gate valve and holds the required pressures for days without additional pumping. The system was inserted into the glovebox where the prepared sample was mounted on the transfer arm, sealed, taken out of the glovebox, and then connected to the XPS instrument. After pumping the mobile system to 5 × 10^−6^ mbar, the sample was transferred on a sample holder and then introduced into the XPS instrument (10^−9^ mbar) *via* a load lock. The data was analyzed by CasaXPS Version 2.3.18PR1.0. Peaks were fitted using LF lineshapes, as implemented in CasaXPS.^11^ For quantification the respective intensities of the Ga 3d and transition metal regions were adjusted by custom sensitivity factors. These sensitivity factors are the product of the specific inelastic mean free paths (TPP-2M equation^12^), the photoionization cross-section,^13^ and a instrument-specific factor. The atomic percentage at% was calculated by dividing these corrected intensities with the sum of all corrected intensities.

The streaming potential measurements have been performed on an improved setup as the one described by Morga *et al.*^14^ Calibration of the streaming potential data has been obtained on muscovite mica (V1 grade, Plano GmbH) in 1 mM KCl solution (BioUltra, Merck) with a titration from pH 3 to pH 10 (HCl/KOH, Titrisol®, Merck). A very good agreement to the data reported by Scales *et al.*^15^ has been found. For the cell a PTFE-spacer with a nominal channel height of 0.3 mm has been used to exclude effects due to surface conductivity. The reported *ζ*-potentials have been calculated by means of the Helmholtz–Smoluchowski approximation. The determination of the isoelectric point (IEP) for the different coatings (SiO_2_, TiO_2_, Al_2_O_3_) has been conducted on mica sheets on which the oxides have been deposited by ALD as described previously. The corresponding measurements have been performed at a constant overall ionic strength of 10 mM (KCl) and titrations from pH 2 to pH 11.

The surface topography of the oxide films on mica has been determined by atomic force microscopy. The AFM measurements have been performed in PeakForce Tapping® mode under ambient conditions (Bruker ICON, Nanoscope V controller). The cantilevers used (ScanAsyst Air, Bruker) have a nominal resonance frequency of 70 kHz and a tip radius <2 nm. The average roughness values reported have been calculated on a scan size of 1 × 1 μm^2^ after plane fit of 1st order.

## Results and discussion

Three main methods for the generation of liquid metal particles have recently been established:

(i) Wet chemical route with thermolysis: the support is impregnated with an organic solution of freshly synthesized (non-purified) gallane–ammine complex (*e.g.* Et_3_NGaH_3_), dried and subsequently submitted to thermal decomposition of the gallane to elementary gallium and gaseous by-products.^16^ However, the utilization of a precursor (Et_3_NGaH_3_) which is not only highly air-sensitive but also unstable at room temperature^17^ hampers a quantitative control of the liquid particles' loading and diameter (distribution).

(ii) Another route is provided by the ultrasonication method. Here, a bulk amount of metallic gallium is dispersed in an organic solvent by ultrasonication. The particle size distribution can be optimized by physical separation techniques (*e.g.* centrifugation). Subsequently, the support is added to this suspension, and the solvent evaporated.^17^

(iii) A broadly applicable route for the synthesis of monodisperse nanocrystals, which is also relevant for the liquid metal particles is the hot injection method. In short, a cold (room-temperature) organometallic precursor solution is injected into a hot (*e.g.* 300 °C) high-boiling apolar coordinating solvent. Upon decomposition of the precursor, the rapid injection results in immediate nucleation of metal particles. The prompt decrease in temperature and concentration then prevents further nucleation and lead to a narrow size distribution *via* exclusive growth. The suspension is finally impregnated into or onto the support.^18,19^

In practice, each approach has its own limitations. Approach (i) can be performed under controlled atmosphere to prevent surface oxidation, but it not designed to allow for quantitative control of the particles size and loading. Approach (iii) delivers the best size distribution control but involves two separate steps, between which a transfer mostly interrupts a perfect inert atmosphere. Approach (ii) relies on a centrifugation step in order to narrow down the size distribution, which prevents an inert-atmosphere handling. In this work, we improve the thermolysis approach and propose a method to generate metallic gallium particles featuring simultaneously an accurate particle size control and minimized oxide layer. For this, we utilize a nanostructured and highly ordered substrate to influence the dewetting that occurs upon decomposition of the originally continuous precursor layer.

**Fig. 1:**
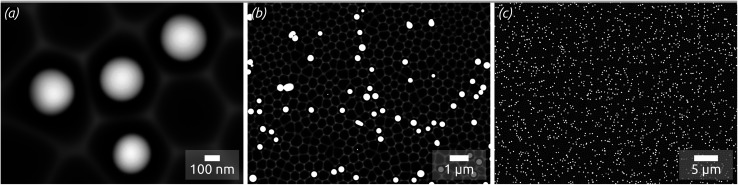
SEM micrographs with distinct magnification levels decreasing from (a–c), taken using a backscattered electron detector (BED, enhancing material contrast): Ga spheres (brighter) formed with the thermal decomposition method using H_3_Ga(quinuclidine) on highly ordered nanostructured substrates consisting of hexagons with a center-to-center distance of approx. 450 nm and indentations depth of approx. 130 nm previously coated with a thin (approx. 10 nm) silica film by atomic layer deposition (ALD).

In concrete terms, a model substrate of overall planar shape but featuring a controlled surface roughness is generated by an one-step electrochemical anodization of aluminium. The anodization process yields indentations in the Al metal foil, forming a hexagonal arrangement with an diameter of approx. 300 nm (Fig. 1). This highly ordered surface nanostructuration serves as a template to tune (i) the spatial separation of the droplets formed and (ii) their size affected by the indentations diameter. Before treatment with a liquid metal precursor, this substrate is coated with the desired oxide surface by atomic layer deposition (ALD), a method allowing for a highly conformal coating of the three-dimensional geometry.^20,21^ With this, the chemical identity of the surface can be adjusted independently of the geometry. For now, let us focus on SiO_2_, as it has been the standard SCALMS substrate so far.^16^ After a toluene solution of quinuclidine–gallane complex has been spin-coated and dried then the resulting layer finally thermally decomposed to volatile by-products and the desired elemental gallium, individual spherical particles nested in the indentations are obtained, as shown in Fig. 1. The high-magnification micrograph, Fig. 1(a), emphasizes how dewetting collects material in each individual indentation, resulting in spheres (droplets above the melting point) that remain separated from one another instead of coalescing. The lower magnifications in Fig. 1(b) and (c) allow one to evaluate how homogeneously the droplets are distributed over a large area. The backscattered electron detector (BED) is used for these micrographs due to its contrast based on materials (elements with higher atomic number, here Ga, appear brighter than Al or Si) instead of the mostly topographic information delivered by a secondary electron detector (SED). We will turn to a quantitative analysis of the Ga particle size distribution and coverage in a later section of this paper. Let us focus on the qualitative materials characterization first.

**Fig. 2:**
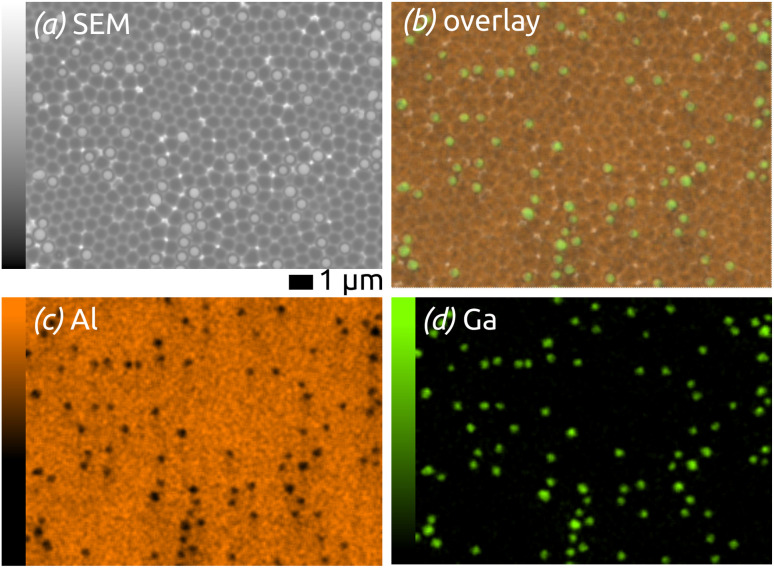
EDX maps of the model SCALMS matrix system: (a) SEM micrograph of the area analyzed; (b) overlay of the Ga and Al signals (in green and orange, respectively) with the SEM micrograph; (c) and (d) individual element maps for Al and Ga.

The elemental composition of the structures is determined by energy-dispersive X-ray microanalysis (EDX, Fig. 2). The chemical composition of the area shown in Fig. 2(a) is mapped in Fig. 2(b–d): on overlay of Al (orange) and Ga (green) signals on the SEM micrograph (b) is complemented with maps of the individual elements. The data prove the successful preparation of well-defined Ga spheres of homogeneous diameter spread and well separated from each other on an ordered, nanostructured SiO_2_/aluminium substrate—an ideal model matrix for SCALMS systems (for which a noble metal still is be added).

**Fig. 3:**
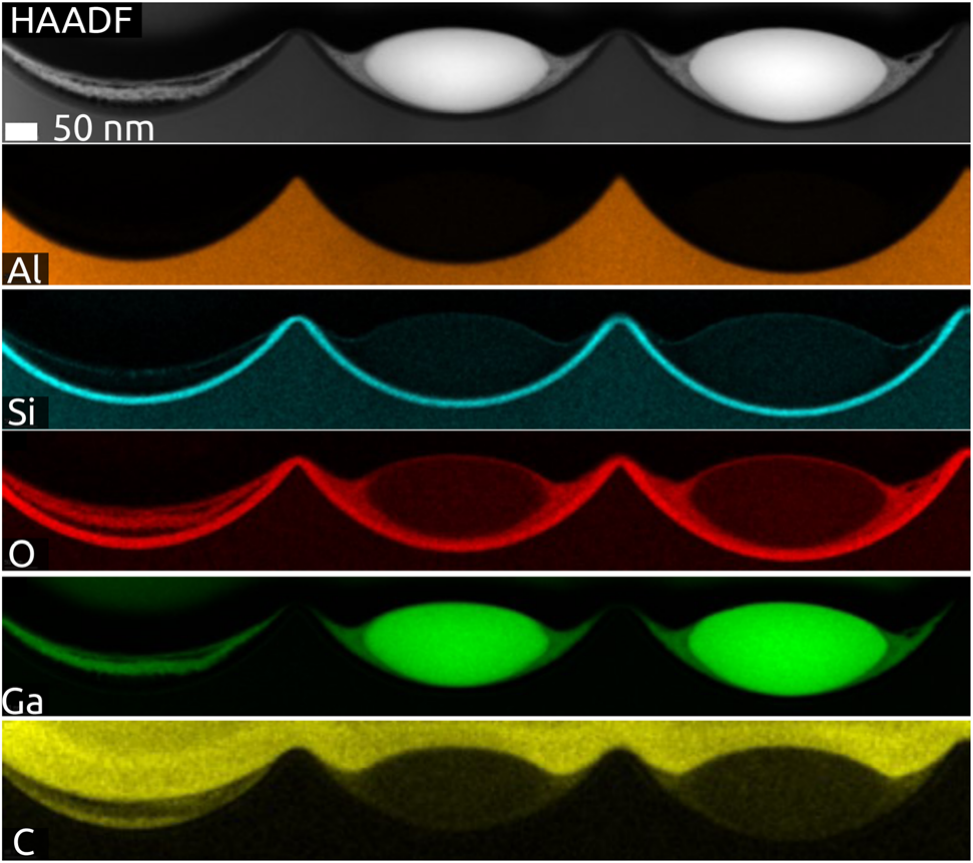
STEM-EDX net intensity mapping of the model SCALMS matrix system in a FIB cross-section with Al (orange), Si (cyan), O (red), Ga (green) and C (yellow) signals: three indentations hosting two Ga particles (HAADF).

The cross-section of a sample after lamella preparation with focused ion beam (FIB) is characterized by scanning transmission electron microscopy (STEM, Fig. 3 and S3 in ESI†). The micrographs and EDX mapping confirm our description above. Firstly, the indentations in Al (orange) have the shape of spherical caps and the SiO_2_ layer has a perfectly constant thickness (cyan Si signal). The Ga particle (green) is in fact imperfectly spherical: it has an ellipsoidal shape instead. Fig. S3 (in ESI)† exhibits small amounts of what seems to be gallium oxide remnants (O signal: red) to the left and right of the metal particle. Fig. 3 confirms that some remnants of gallium oxide are left over from the quinuclidine gallane precursor film in the indentations unoccupied by Ga metal. In other words, imperfectly anaerobic conditions cause some of the material to be oxidized and some of the Ga contents are lost inasmuch as they do not contribute to the metal particles. The high reactivity of Ga to oxidation also causes the formation of a thin gallium oxide layer upon sample transfer. Its thickness is approximately 5 to 6 nm. This is in stark contrast to the significant oxidation observed usually, with tens if not hundreds of nm of oxide. In application, this oxide is reduced in conditions of catalysis (elevated temperature and hydrogen pressure), but it influences the size and shape of the SCALMS particles, as well as their mobility and agglomeration, in an uncontrolled manner. The near-pristine state obtained here will minimize this source of uncertainty.

**Fig. 4:**
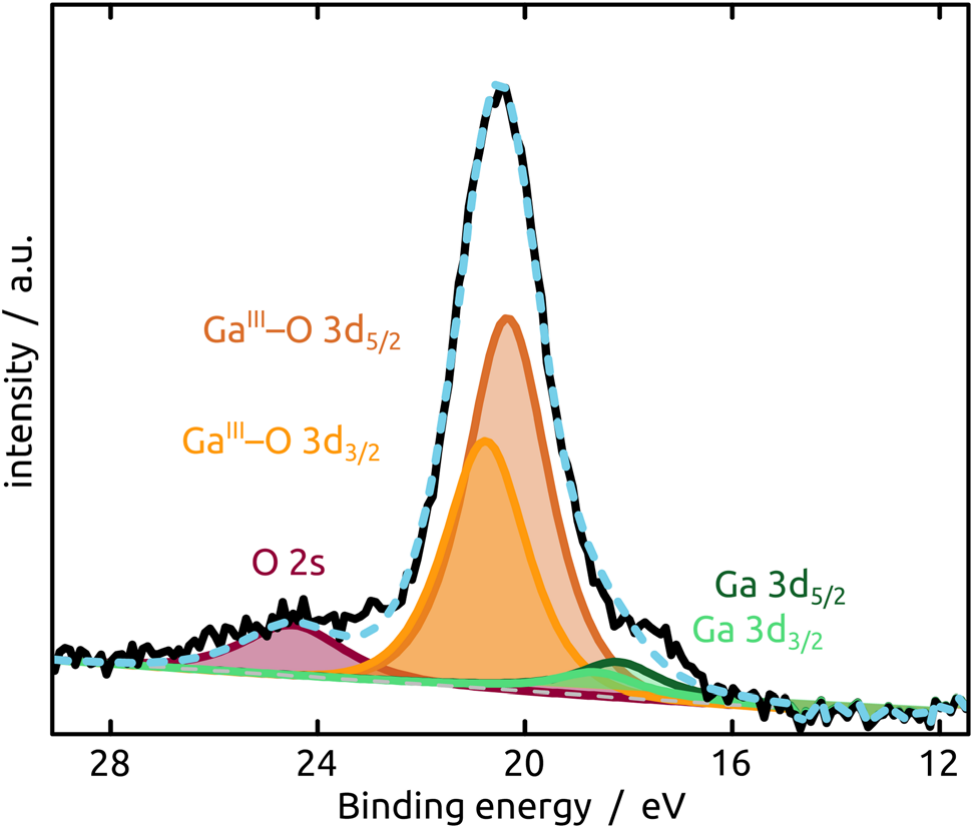
XPS analysis of the model SCALMS matrix system indicating a passivating gallium oxide layer at the surface of the metallic Ga particle.

The generation and existence of a (liquid) elementary Ga core in the particles on the nanostructured surface is further confirmed by X-ray photoelectron spectroscopy (XPS, Fig. 4). Although the extreme surface sensitivity of the method yields a predominant signal of Ga_2_O_3_ (Ga 3d doublet at approx. 20.3 eV and 20.8 eV with *Δ* = 0.42 eV, O 2s at approx. 24.5 eV), a Ga 3d doublet at 18.2 eV and 18.6 eV with *Δ* = 0.42 eV is visible and assigned to metallic Ga buried below the thin passivation layer formed on the surface.^22^ In the experimental conditions of our XPS measurements, the probe depth is approximately 9 nm, indicating that the oxide layer thickness is slightly below this value. This is in perfect agreement with the value of 5–6 nm determined by STEM above.

X-ray diffraction measured in grazing incidence geometry (GI-XRD, Fig. S1 and S2 in ESI†) does not provide any direct evidence for the presence of crystalline Ga or Ga_2_O_3_. The diffractograms most prominently display the signals of the Al substrate at 2*θ* values 38°, 45°, 65° and 78°, with additional small peaks for the ALD-deposited oxide, if that is crystalline (as in the case of TiO_2_).

**Fig. 5:**
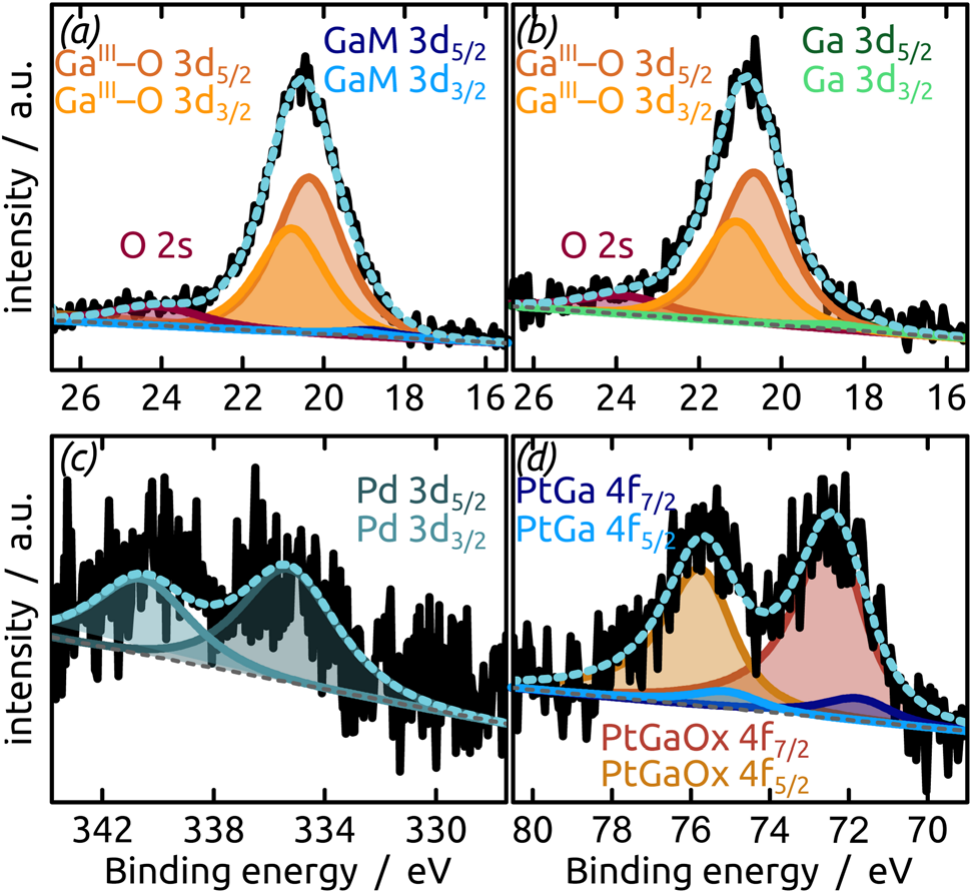
XPS analysis ((a and b) Ga region; (c and d) noble metal region) of the model SCALMS system after partial transmetallation with a noble metal ((a and c) Pd; (b and d) Pt) from solution under air. The data indicate incorporation of the noble metals in the Ga-based particle (oxidized at its surface).

The final preparation step towards a functional SCALMS catalyst is the incorporation of the active noble metal in a small concentration in the Ga matrix. Fig. 5 demonstrates that our model system successfully incorporates either Pt or Pd as desired upon partial transmetallation with mixed (water/isopropanol) solutions of the corresponding Pt(iv) or Pd(ii) precursors. The partial galvanic exchange of Ga particles with Pd leads to a system in which the binding energy of the palladium indicates a Pd^0^ (Pd 3d doublet at 340.6 eV and 335.3 eV with spin–orbit separation of *Δ* = 5.26 eV). However, a mixture of a GaPd alloy and Pd^0^ seems likely due to the broad signal.^23^ The exchange of Ga with Pt leads to the formation of a GaPt intermetallic compound (IMC, PtGa 4f doublet at 74.3 eV and 71.0 eV with *Δ* = 3.33 eV),^24^ as expected from the phase diagram of GaPt.^25^ The main signal in the Pt 4f region results from Pt incorporated into gallium oxide, where Pt is positively charged.^26,27^ A similar positive charge on Pt has been observed for PtGa systems in the past, as well as during the oxidation of GaRh SCALMS. In the latter case, DFT simulations and calculations show Bader charges of approx. +1.25*e* for Rh in Ga_2_O_3_ (ref. 28) (PtGa oxide 4f doublet at 75.7 eV and 72.4 eV with *Δ* = 3.32 eV).^26,27^

The XPS signals of Pt or Pd appear in low concentration as desired (1.5 at% transition metal), but incorporated in GaO_*x*_ due to the aerobic treatment. Thus, the data demonstrate that our geometrically near-perfect model system is adequate for studies of SCALMS catalysis. Let us now turn to the effect of the underlying surface chemistry.

**Fig. 6:**
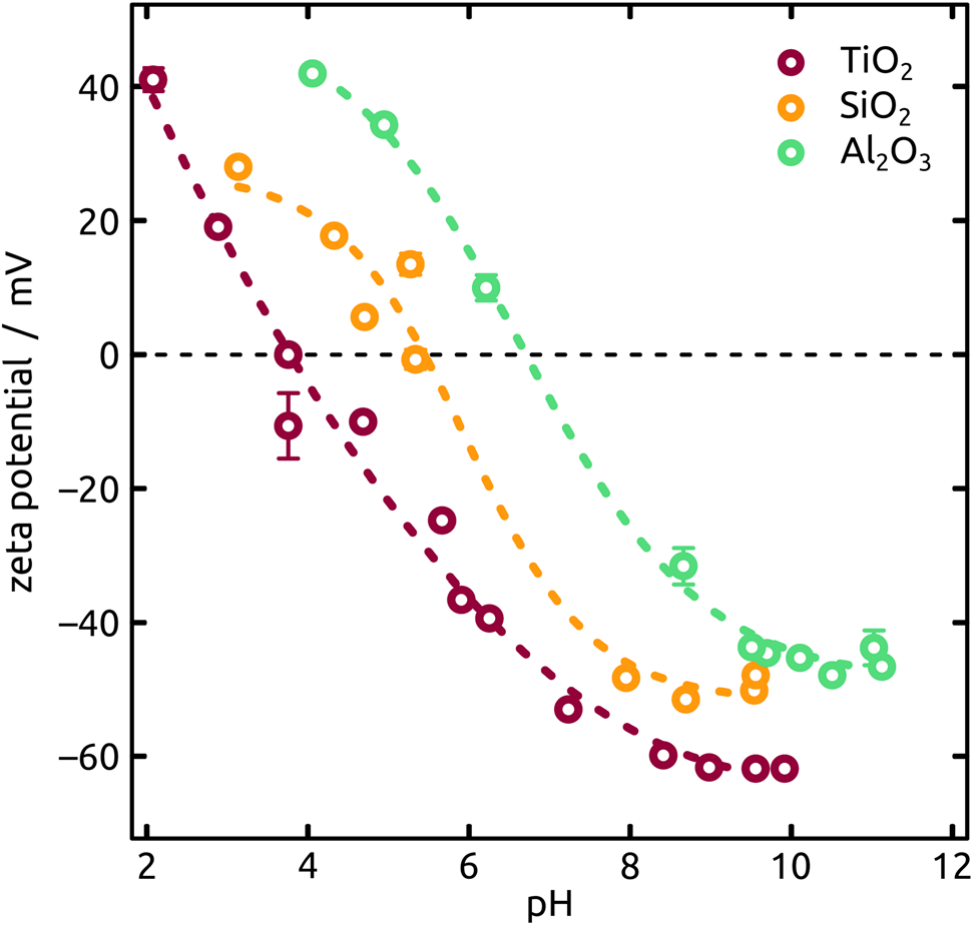
Characterization of the IEP (ionic strength of 10 mM KCl and titrations from pH 2 to pH 11) of distinct metal oxides deposited on mica sheets by ALD (thicknesses in the range of approx. 10 nm to 15 nm).

An increasing body of catalysis literature shows that even in redox catalysis, not only the redox-active noble metal but also the acid–base interaction with the underlying oxide substrate is of relevance.^29,30^ Let us investigate, accordingly, how the oxide layer present on the Al indentations affects the distribution of Ga particles, going from acidic oxides (TiO_2_, SiO_2_) to amphoteric (Al_2_O_3_) and basic (MgO) ones. The surface chemistry can affect both the distribution of gallane precursor film upon spin coating and the subsequent dewetting of the Ga layer generated by thermal decomposition. The quantitative parameters which we will consider here are the particle diameter (and the diameter distribution) and the number of particles per unit area (particle density). We will correlate them with the isoelectric point (IEP), as defined by the pH value at which a certain oxide in contact with an aqueous solution has equal density of positive (protonated) and negative (deprotonated) surface sites (Table 1). The IEP determined by streaming potential measurements for the amorphous, partially hydrated ALD thin films (Fig. 6) exhibit slight deviations from the values commonly accepted for the corresponding bulk materials. The data presented in the table are obtained by automatic image analysis of BED-SEM micrographs, which provides a number of bright particles and total surface of bright pixels as the raw data. The data are then converted to particle density and diameter distribution based on the following assumptions and procedures: (i) spherical shape assumption to calculate diameter from area, (ii) known micrograph area to calculate the particle density as number of particles per unit area (1 μm^2^), and (iii) the coverage factor as ratio between bright pixels and total pixel number. These numbers also allow us to calculate an equivalent Ga layer thickness, corresponding to the thickness of a hypothetical Ga film homogeneously covering the substrate with the same total amount of Ga as actually present in the particles.

**Table 1:** Summary of quantitative geometric Ga particle parameters obtained for oxides with various isoelectric points (IEP). We provide IEP values related to the bulk solids from the literature^31^ and experimental ones obtained by streaming potential measurements (apart from MgO, which was insufficiently stable for reliable measurements). Particle diameter *∅* and *N* quantifies the sample size (total number of particles counted). *SE of mean: standard error of mean; **range: defined as the interquartile range (Q3–Q1)

Oxide	IEP bulk mat.	IEP thin film	*∅* ± SE of mean*/nm	Range** nm	*N*
TiO_2_	4–7 (ref. 32–35)	3–4	235 ± 6	154	414
SiO_2_	<3 (ref. 36–39)	5–6	284 ± 1	38	6229
Al_2_O_3_	8 (ref. 40 and 41)	6–7	335 ± 31	332	55
MgO	11	n/a	440 ± 10	202	232

**Fig. 7:**
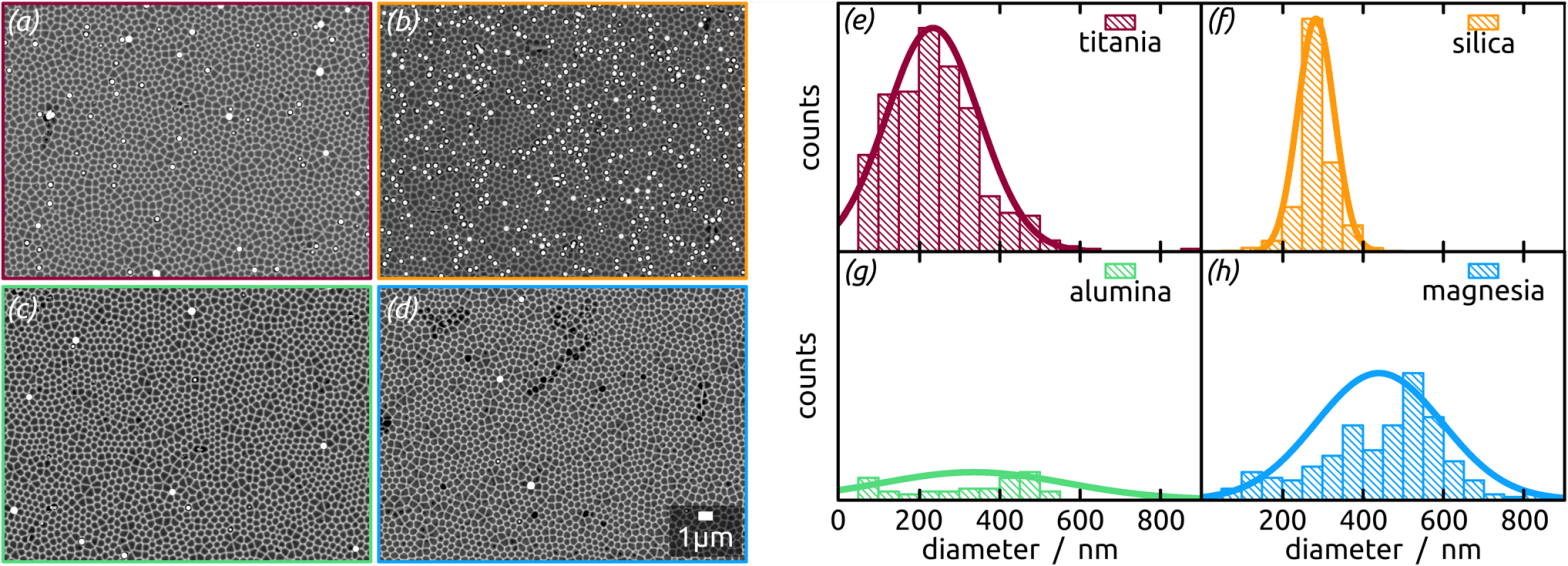
(a–d) SEM micrographs (BED) of Ga particles (brighter due to higher atomic number) formed with the thermal decomposition method using H_3_Ga(quinuclidine) on highly ordered nanostructured substrates previously coated with distinct metal oxide thin films by ALD, and (e–h) corresponding histogram of diameter distributions with the normal distribution fits: (a) and (e) TiO_2_, (b) and (f) SiO_2_, (c) and (g) Al_2_O_3_ and (d) and (h) MgO.

The Ga particles on substrates coated with more acidic oxides (TiO_2_: Fig. 7(a); SiO_2_: Fig. 1 and 7(b)) yield the smallest average diameters, followed by the amphoteric (Al_2_O_3_) and basic (Mg) ones, Fig. 7. In fact, we observe an approximately linear increase of particle diameter with IEP, Fig. S5 (in ESI,† using the literature value for IEP of MgO). This trend (increase by a factor 2 approximately) is roughly accompanied by an even steeper, albeit less monotonous, broadening of the size distribution (by a factor 50 approximately), whereby the narrowest distribution is obtained not for TiO_2_ but for SiO_2_ instead.

**Fig. 8:**
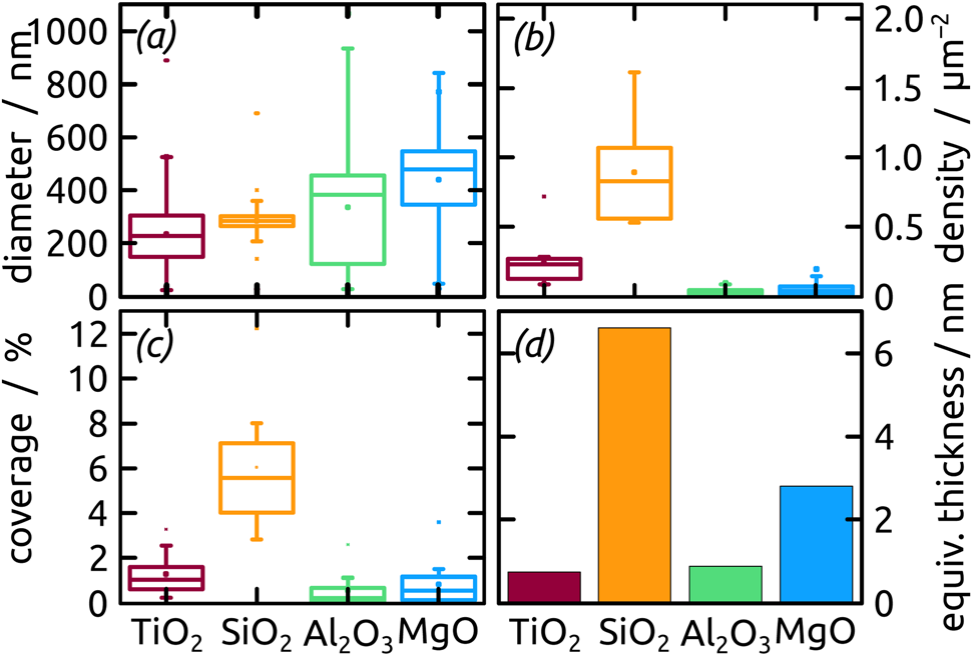
Box–whisker-plot of particle (a) diameter, (b) density, (c) coverage distribution and (d) equivalent thickness of Ga on distinct metal oxides with different surface chemistry.

The micrographs evidence in obvious manner that samples featuring larger particles also exhibit a lower particle density, which is intuitive. Are the two parameters inversely proportional to each other to conserve mass? To address this question, we summarize all three quantitative parameters in the box–whisker-plot, Fig. 8, together with a quantification of the total Ga amount present on each sample, namely the equivalent Ga thickness. This way of presenting the results emphasizes that the various oxides not only influence the particle size distribution (by affecting the dewetting) but also the absolute amount of material present (that is, they already influence film formation during spin coating of the precursor solution). The surfaces best able to fix quinuclidine–gallane are the most extreme ones on the IEP range, perhaps because their Lewis acid-based reactivity complements that of the Lewis acid–base complex ideally. The surface best able to prevent large motions of metallic Ga and coalescence to large particles are the acidic ones, probably due to a lower interfacial energy.

In summary, the highest degree of control in terms of diameter distribution (smallest average Ga particle diameter and narrowest size distribution both in absolute and relative terms), the highest density (highest number of Ga particles per unit area) and thus the highest areal and mass coverage are all achieved with SiO_2_ as the substrate.

## Conclusions

The results presented here establish the viability of a quasi-planar SCALMS model system offering a high degree of geometric control over the metal particles. The manufacturing procedure combines anodization as a nanostructuration method to generate highly controlled surfaces with ALD to adjust the substrates' surface chemistry independently of their geometry. The generation of elementary gallium droplets with well-defined diameter and size distribution by spin coating and subsequent thermal decomposition approach under inert atmosphere offers the possibility to prepare Ga as the liquid matrix material without the generation of a fully oxidized material as an intermediate or ‘pre-catalyst’. Using indentations with a center-to-center distance of approx. 450 nm and indentations depth of approx. 130 nm coated with silicon dioxide yields a SCALMS model featuring Ga ellipsoids of *ca.* 280(±40) nm. These are still rather large particles in the catalysis field overall, but much smaller than the extremely wide size distributions available currently, which reach the >μm range. Furthermore, the preparative methods used offer a variety of experimental parameters that can be varied in order to optimize the system to any given application.

In the coming months, we will exploit this system to perform detailed kinetic investigations of SCALMS catalysis, hopefully enabling us to determine for the first time values such as the turnover frequencies. In parallel, we will study experimentally and theoretically interfacial energies in the metal oxide/Ga metal system in order to shed light onto the dewetting phenomena at work in SCALMS catalysis. Mastering them will be of utmost importance for the efficient use of every noble metal atom.

## Author contributions

André Hofer: conceptualization, methodology, investigation, visualization, writing – original draft. Nicola Taccardi: investigation. Michael Moritz: investigation. Christoph Wichmann: investigation. Sabine Hübner: investigation. Dominik Drobek: investigation. Matthias Engelhardt: investigation. Georg Papastavrou: validation, resources. Erdmann Spiecker: validation, resources, funding acquisition. Christian Papp: validation, resources, funding acquisition. Peter Wasserscheid: conceptualization, validation, resources, project administration, funding acquisition. Julien Bachmann: conceptualization, resources, supervision, writing – review & editing, validation, funding acquisition.

## Conflicts of interest

There are no conflicts to declare.

## Supplementary Material

RA-013-D2RA07585G-s001
