# Supplementary material for: Preparation of geometrically highly controlled Ga particle arrays on quasi-planar nanostructured surfaces as a SCALMS model system
Source: RSC Adv. 2023 Jan 27;13(6):4011–8. doi: 10.1039/d2ra07585g (PMC9890623; doi:10.1039/d2ra07585g)
Supplement: RA-013-D2RA07585G-s001 [file RA-013-D2RA07585G-s001.pdf]

Electronic Supplementary Material (ESI) for RSC Advances.
This journal is © The Royal Society of Chemistry 2023

Preparation of geometrically highly controlled Ga particle arrays on quasi-planar nanostructured surfaces as a SCALMS model system

André Hofer,[a] Nicola Taccardi,[b] Michael Moritz,[c] Christoph Wichmann,[c] Sabine Hübner, [d] Dominik Drobek,[d] Matthias Engelhardt,[e] Georg Papastavrou,[e] Erdmann Spiecker,[d] Christian Papp,[c,f] Peter Wasserscheid,[b,g] and Julien Bachmann[a]*

Contents

1 **Grazing incidence x-ray crystallography** 2

2 **Scanning transmission electron microscopy** 3

3 **Atomic force microscopy** 3

4 **Characterization of the isoelectric point** 4

5 **Automated SEM micrograph analysis** 4

[a] *Friedrich-Alexander-Universität Erlangen-Nürnberg (FAU), Department of Chemistry and Pharmacy, Chemistry of Thin Film Materials, IZNF, Cauerstr. 3, 91058 Erlangen, Germany; *E-mail: julien.bachmann@fau.de*

[b] *Friedrich-Alexander-Universität Erlangen-Nürnberg (FAU), Department Chemical and Biological Engineering, Institute of Chemical Reaction Engineering (CRT), Egerlandstr. 3, 91058 Erlangen, Germany*

[c] *Friedrich-Alexander-Universität Erlangen-Nürnberg (FAU), Department of Chemistry and Pharmacy, Chair of Physical Chemistry II, Egerlandstr. 3, 91058 Erlangen, Germany*

[d] *Friedrich-Alexander-Universität Erlangen-Nürnberg (FAU), Institute of Micro- and Nanostructure Research (IMN) & Center for Nanoanalysis and Electron Microscopy (CENEM), IZNF, Cauerstr. 3, 91058 Erlangen, Germany*

[e] *Universität Bayreuth, Chair of Physical Chemistry II, Universitätsstr. 30, 95447 Bayreuth, Germany*

[f] *Angewandte Physikalische Chemie, FU Berlin, Arnimalle 22, 14195 Berlin*

[g] *Forschungszentrum Jülich GmbH, Helmholtz Institute Erlangen-Nürnberg for Renewable Energy (IEK-11), Cauerstr. 1, 91058 Erlangen, Germany*

1 Grazing incidence x-ray crystallography

The crystal structure of the Ga matrix for a SCALMS model system was analysed by GI-XRD. The pattern of the particles on nanostructured substrates previously coated with either silica (Fig. S1) or titania (Fig. S2) by ALD indicates predominantly the Al (2θ: 38 °, 45 °, 65 ° and 78 °) signal of the support.

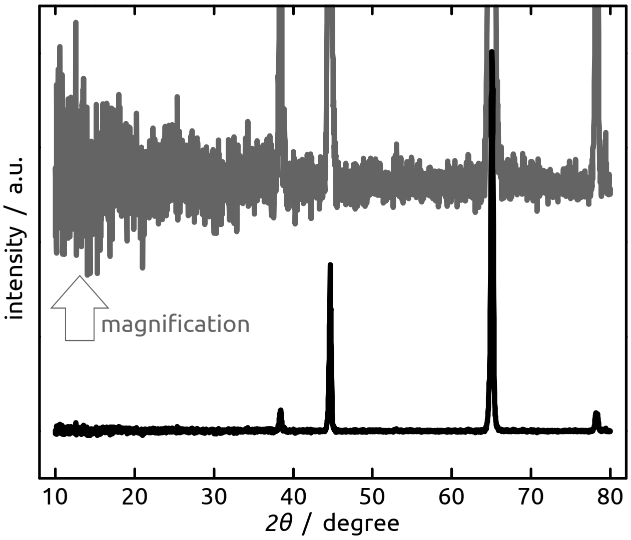

Fig. S1 GI-XRD pattern of of SCALMS matrix model system: Ga particles on silica coated nanostructured indentations indicating predominantly Al (2θ: 38 °, 45 °, 65 ° and 78 °) signal of the substrate material.

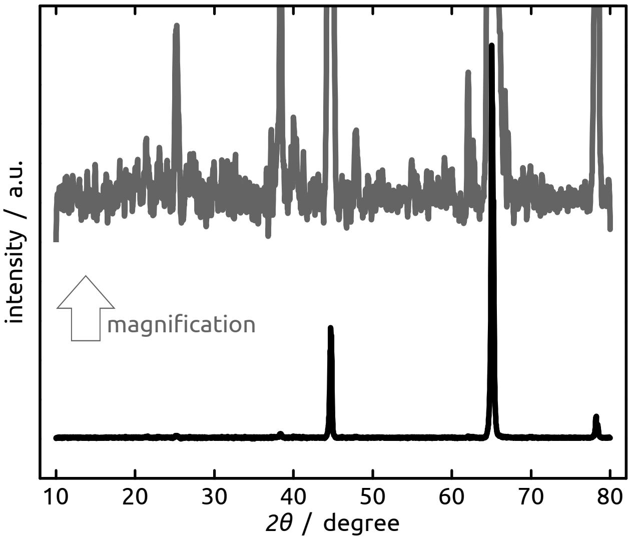

Fig. S2 GI-XRD pattern of of SCALMS matrix model system: Ga particles on titania coated nanostructured indentations indicating TiO_2 in anatase structure, and a predominantly Al (2θ: 38 °, 45 °, 65 ° and 78 °) signal of the substrate material.

2 Scanning transmission electron microscopy

The cross-section of a sample after lamella preparation with focused ion beam (FIB) is characterized by scanning transmission electron microscopy (STEM, Figure S3). The indentations in Al (orange) have the shape of spherical caps and the SiO_2 layer has a perfectly constant thickness (cyan Si signal). The Ga particle (green) is in fact imperfectly spherical: it has an ellipsoidal shape instead. Fig. S3 exhibits small amounts of what seems to be gallium oxide remnants (O signal: red) to the left and right of the metal particle.

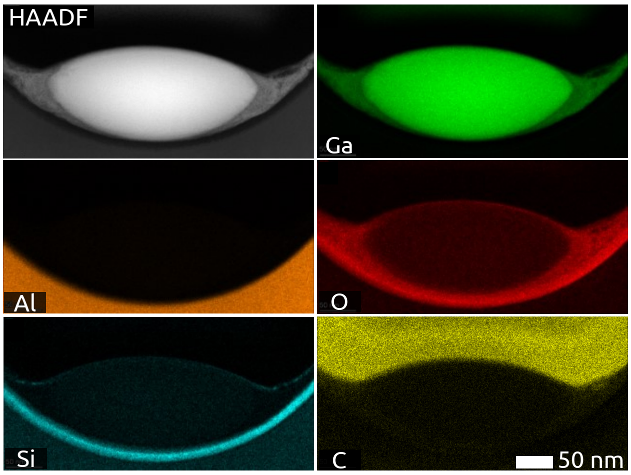

Fig. S3 STEM-EDX net intensity mapping of the model SCALMS matrix system in a FIB cross-section with Ga (green), Al (orange), O (red), Si (cyan) and C (yellow) signals: single indentation with Ga particle (HAADF).

3 Atomic force microscopy

Atomic force microscopy (AFM) characterizations were performed previously the streaming potential measurements to determine the roughness R_a of distinct metal oxide coated mica sheets. The oxides were deposited by atomic layer deposition (ALD). Figure S4 presents the topography of an approx. 1 μm x 1 μm area: *(a)* TiO_2 with a R_a = 0.2 nm (at flat spots), *(b)* SiO_2 with a R_a = 0.2 nm and *(c)* Al_2O_3 with a R_a = 0.2 nm. The pristine mica sheets exhibits a R_a = 0.1 nm after the streaming potential experiment (not shown).

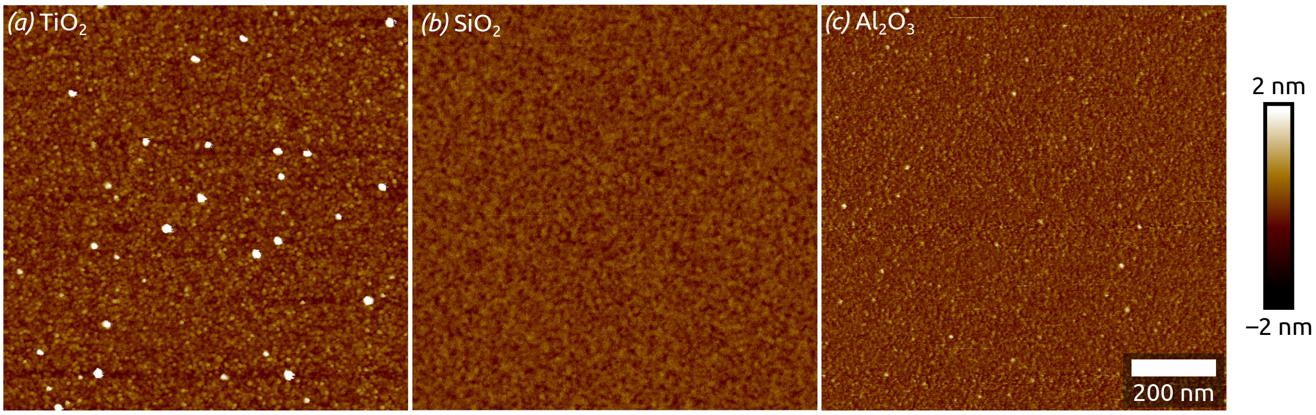

Fig. S4 Atomic force microscopy (AFM) roughness R_a characterization of distinct metal oxide coated mica sheets presenting the topography of an approx. 1 μm x 1 μm area: *(a)* TiO_2, *(b)* SiO_2 and *(c)* Al_2O_3.

4 Characterization of the isoelectric point

The IEP determined by streaming potential measurements for the amorphous, partially hydrated ALD thin films. The isoelectric point (IEP) is defined by the pH value at which a certain oxide in contact with an aqueous solution has equal density of positive (protonated) and negative (deprotonated) surface sites. An approximately linear increase of particle size diameter related to experimentally determined IEP values of the various metal oxide films (exception: literature value[1] used for MgO) can be observed.

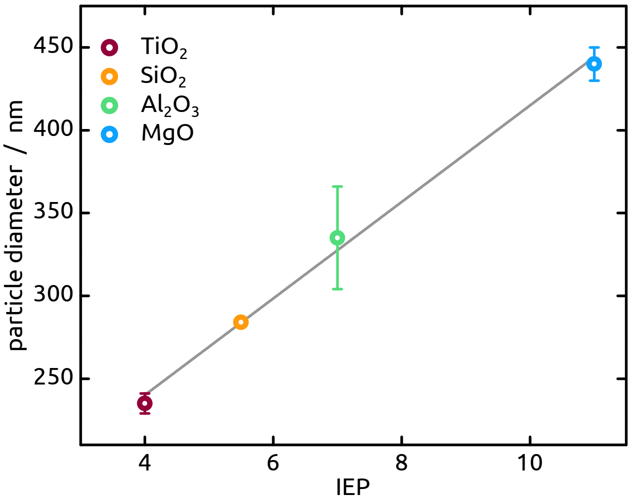

Fig. S5 Particle size diameter related to experimentally determined IEP values of the various metal oxide films (exception: literature value[1] used for MgO).

5 Automated SEM micrograph analysis

The Ga particle size is analysed by automated image-analysis (software: ImageJ, v1.53k). SEM-micrographs obtained using a SE-detector Fig. S6 *(a)* are detrimental due to morphology enhanced contrast. Contrast and brightness adjusted micrographs utilizing the BE-detector (enhancing material contract, Fig. S6 *(b)*) are used instead for further analysis. The Ga particles appear in brighter pixels due to their higher atomic number than the substrate elements. Subsequent threshold adjustment (Fig. S6 *(c)*) yields to the format from which the particle size can be recognized and analyzed automatically (Fig. S6 *(d)*).

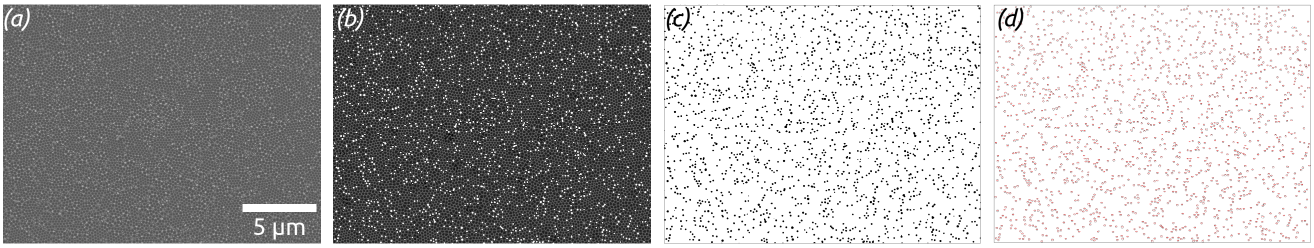

Fig. S6 Automated SEM-mirograph analysis using ImageJ, v1.53k.

References

1 M. Kosmulski, *J. Colloid Interface Sci.*, 2002, **253**, 77–87.
